# Automated matching of two-time X-ray photon correlation maps from phase-separating proteins with Cahn–Hilliard-type simulations using auto-encoder networks

**DOI:** 10.1107/S1600576722004435

**Published:** 2022-06-15

**Authors:** Sonja Timmermann, Vladimir Starostin, Anita Girelli, Anastasia Ragulskaya, Hendrik Rahmann, Mario Reiser, Nafisa Begam, Lisa Randolph, Michael Sprung, Fabian Westermeier, Fajun Zhang, Frank Schreiber, Christian Gutt

**Affiliations:** a Department Physik, Universität Siegen, Walter-Flex-Strasse 3, 57072 Siegen, Germany; b Institut für Angewandte Physik, Universität Tübingen, Auf der Morgenstelle 10, 72076 Tübingen, Germany; c Department of Physics, AlbaNova University Center, Stockholm University, SE-106 91 Stockholm, Sweden; d Deutsches Elektronen-Synchrotron DESY, Notkestraße 85, 22607 Hamburg, Germany; Charles University, Prague, Czech Republic and CEITEC at Masaryk University, Brno, Czech Republic

**Keywords:** protein dynamics, X-ray photon correlation spectroscopy, XPCS, Cahn–Hilliard, auto-encoders, machine learning

## Abstract

Machine learning methods are used for an automated classification of experimental two-time X-ray photon correlation maps from an arrested liquid–liquid phase separation of a protein solution. The correlation maps are matched with correlation maps generated with Cahn–Hilliard-type simulations of liquid–liquid phase separations according to two simulation parameters and in the last step interpreted in the framework of the simulation. The matching routine employs an auto-encoder network and a differential evolution based algorithm. The method presented here is a first step towards handling large amounts of dynamic data measured at high-brilliance synchrotron and X-ray free-electron laser sources, facilitating fast comparison with phase field models of phase separation.

## Abstract 2

Two-time correlation maps are classified in a simulation framework using an auto-encoder network.

## 1. Introduction

X-ray photon correlation spectroscopy (XPCS) is an experimental method capable of accessing the dynamics of protein systems over length scales that range from the atomic to micrometre scale, and timescales from microseconds to hours (X. Lu *et al.*, 2008 ▸; Zhang *et al.*, 2011 ▸, 2017 ▸; Madsen *et al.*, 2016 ▸; Möller *et al.*, 2016 ▸; Zinn *et al.*, 2018 ▸; Perakis & Gutt, 2020 ▸; Ruta *et al.*, 2012 ▸; Begam *et al.*, 2021 ▸). In XPCS, time series of coherent X-ray speckle patterns are measured, giving access to dynamical properties via time-resolved correlation maps – the two-time correlation function (TTC) (Brown *et al.*, 1997 ▸; Bikondoa, 2017 ▸). With fast megapixel 2D X-ray detectors, XPCS experiments can produce large quantities of data in a short time interval with up to thousands of TTCs per hour of beamtime. Given the typical duration of a synchrotron experiment of a few days, the resulting quantities of TTCs are difficult to handle. Therefore, there is a generic need for methods facilitating fast and reliable analysis including classification of the data. This is especially important with regards to steering, selecting and controlling the experimental parameters during the beamtime. However, in the aftermath of the experiment, quick classification methods are important for benchmarking models which, for example, simulate a gel transition or the solidification process of a protein solution upon liquid–liquid phase separation (LLPS).

Phase separation is a ubiquitous process in nature, with applications and consequences for a wide range of scientific disciplines such as solid-state physics, material sciences and biology. Phase separation in biological systems attracted considerable attention with the discovery that LLPS in protein solutions (Ishimoto & Tanaka, 1977 ▸; Schurtenberger *et al.*, 1989 ▸; Broide *et al.*, 1991 ▸; Berland *et al.*, 1992 ▸; Muschol & Rosenberger, 1997 ▸) constitutes a possible pathway for organizing membraneless structures in living cells (Brangwynne *et al.*, 2009 ▸; Shin & Brangwynne, 2017 ▸; Berry *et al.*, 2018 ▸). Detailed investigations were carried out with respect to the biological functions of these protein condensates, encompassing biochemical reaction rates, buffering protein concentrations, and sensing or signaling (Shin & Brangwynne, 2017 ▸). A variety of diseases which result from a loss or change of function of these condensates are accompanied by phase transitions (Malinovska *et al.*, 2013 ▸; Weber & Brangwynne, 2012 ▸).

Phase separation is initiated by quenching a system into the metastable region of the phase diagram, launching a process of out-of-equilibrium self-organization (Dong & Granick, 2021 ▸). The state of the condensates depends on the dynamic and kinetic processes during their formation, with dynamical asymmetries between the two phases on a hierarchy of length and timescales and invoking viscoelastic properties of the resulting network structures (Berry *et al.*, 2018 ▸; Zaccarelli, 2007 ▸; Tanaka, 2000 ▸). In the biological context, an understanding of the component timescales is important as they are expected to meet the intrinsic timescales of biochemical processes in the condensates.

Upon phase separation, the dynamics slow down on mol­ecular length scales due to local concentration changes. This microscopic deacceleration can eventually lead to the arrest of phase separation on larger length scales complemented by the formation of bicontinuous gel network structures – as observed in colloidal and protein systems (Manley *et al.*, 2005 ▸; Conrad *et al.*, 2010 ▸; P. J. Lu *et al.*, 2008 ▸). Employing time-resolved scattering experiments, the kinetics during arrested phase separations have been observed frequently as a slowdown of the growth of the static structure factor *S*(*Q*) in both *Q* position and peak height for low-temperature quenches (Gibaud & Schurtenberger, 2009 ▸; Gibaud *et al.*, 2011 ▸; Cardinaux *et al.*, 2007 ▸; Da Vela *et al.*, 2016 ▸, 2017 ▸, 2020 ▸; Bucciarelli *et al.*, 2015 ▸). In contrast, the dynamics of protein solutions evolving into an arrested phase transition are still poorly understood as studies require the concurrent monitoring of an extraordinarily broad range of length and timescales. Likewise, experimental validation of models of the dynamics of critical phenomena during LLPS like the Cahn–Hilliard equation (CHE) (Cahn, 1965 ▸) and other correlated models is still needed, particularly with respect to glass–gel transitions displaying considerable dynamical asymmetries between the dilute and concentrated phase (Berry *et al.*, 2018 ▸; Girelli *et al.*, 2021 ▸; Ragulskaya *et al.*, 2021 ▸).

Machine learning (ML) has already proven to be useful in the analysis of various structural X-ray data from small-angleX-ray scattering (Archibald *et al.*, 2020 ▸; Chen & Pollack, 2020 ▸; Franke *et al.*, 2018 ▸; Wang *et al.*, 2017 ▸) and wide-angle X-ray scattering (Chen & Pollack, 2020 ▸; Wang *et al.*, 2017 ▸), diffraction (Berntson *et al.*, 2003 ▸; Oviedo *et al.*, 2019 ▸; Vecsei *et al.*, 2019 ▸), and reflectometry (Greco *et al.*, 2019 ▸) experiments. The algorithms provide real-time feedback (Wang *et al.*, 2017 ▸; Ke *et al.*, 2018 ▸; Greco *et al.*, 2019 ▸) during the experiment or identify and remove bad images to reduce the stored data volume (Wang *et al.*, 2017 ▸; Ke *et al.*, 2018 ▸). In many cases the ML algorithms have been trained with synthetic structural data generated from existing databases on proteins (Franke *et al.*, 2018 ▸; Archibald *et al.*, 2020 ▸), RNA (Chen & Pollack, 2020 ▸) or crystal structures (Oviedo *et al.*, 2019 ▸; Vecsei *et al.*, 2019 ▸), which enables them to classify the results obtained in experiments without an expert spending time on labeling large quantities of data for training (Berntson *et al.*, 2003 ▸). Recently, ML algorithms have also been trained to reduce the noise of experimental TTCs (Konstantinova *et al.*, 2021 ▸). The CHE has also been used in different ML tasks (Wight & Zhao, 2021 ▸; Pokuri *et al.*, 2019 ▸; Zhang & Garikipati, 2020 ▸; Farimani *et al.*, 2018 ▸).

The focus of X-ray scattering applications of ML has been mainly on static properties up to now. We note, however, that many processes in nature, such as the LLPS of protein solutions, display dynamic phenomena which require the development of schemes and methods capable of classifying the corresponding dynamic X-ray signatures of the processes. ML-based classification of process data evolving in time is computationally very expensive due to the large quantity of training data required. Employing a minimum 2D model which allows a network with finite computer resources to be trained, we report on a first step towards this goal and present a neural network method for analysis and classification of dynamical X-ray information during LLPS.

The CHE in a simplified 2D version still captures the essence of the physics involved (Sciortino *et al.*, 1993 ▸; Sappelt & Jäckle, 1997 ▸; Lamorgese & Mauri, 2009 ▸), while allowing us to generate a large number of simulations on the timescale of hours, and couple it with a concentration-induced gelation as proposed by Sciortino *et al.* (1993 ▸) and Sappelt & Jäckle (1997 ▸). We aim for an automated assignment of the measured TTCs in the work of Girelli *et al.* (2021 ▸) with TTCs from simulations that differ in the parameters ε, *i.e.* proportional to reduced quench depth, and 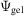 , *i.e.* the order parameter at which the mobility drops by a factor of 1/2. We train an auto-encoder neural network with TTC simulation data and employ a differential evolution based algorithm for matching encoded experimental TTCs with simulations. Our work constitutes a first step to making use of the static, kinetic and dynamical information contained in XPCS data for ML-based analysis of processes in phase separation.

## 2. Analysis and results

### 2.1. Simulation of the Cahn–Hilliard equation

We model the dynamic processes during the spinodal decomposition with the help of the CHE (Sciortino *et al.*, 1993 ▸; Gunton *et al.*, 1983 ▸), 

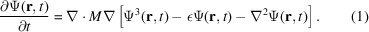

**Figure 1:**
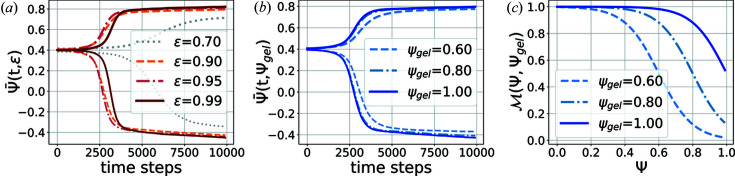
(*a*), (*b*) Order parameter averaged over all lattice sites having an order parameter higher/lower than the initial order parameter ( 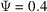 ) as a function of time. In (*a*), 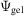 was fixed to 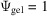 and ε was varied. For 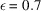 , the phase separation is much slower and the concentration of the dense phase is lower at the end of the chosen time window. This makes it hard to see the effects of high 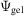 , which is why ε is restricted to 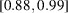 for the generation of the simulated training data. In (*b*), ε was fixed to 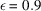 and 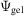 was varied. The phase separation starts later for larger values of ε due to the smaller initial fluctuations. For constant ε, the phase separations show similar behavior at early times but the droplet formation is faster for larger values of 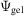 . (*c*) Mobility as a function of the order parameter and the gelation point 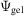 marking the order parameter at which the mobility drops to 0.5.

Here 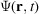 is the order parameter at a given time *t* and position 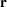 which is related to the local protein concentration Φ by 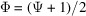 (Sciortino *et al.*, 1993 ▸). The symmetry breaking is introduced by the parameter ε which can be identified as being proportional to the reduced quench depth 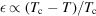 , where 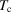 is the upper critical temperature of the system. If ε fulfills the condition 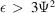 (*e.g.* Gunton *et al.*, 1983 ▸), the system is in an unstable state below the spinodal curve and separates into regions with higher and lower local protein concentrations [Figs. 1 ▸(*a*) and 1 ▸(*b*)].

The phase separation by spinodal decomposition is coupled to a gelation process by introducing a concentration-dependent mobility parameter *M* in equation (1) (Sappelt & Jäckle, 1997 ▸) which reduces the mobility of the highly concentrated phase via

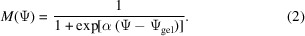

In this model the mobility decreases from values 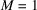 to 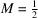 when the order parameter Ψ exceeds the value of 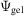 [Fig. 1 ▸(*c*)]. The parameter α is fixed to 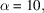 providing a steep decrease of the mobility as required for an arrested phase separation (Sappelt & Jäckle, 1997 ▸).

We perform the simulation on a 2D grid of size 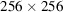 . The grid uses periodic boundary conditions and is initialized with a mean order parameter of 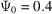 such that we observe droplets with a low concentration instead of an interconnected network, which would be the case for 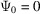 . The fluctuations necessary for LLPS are added as random noise with a maximum amplitude of 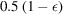 . Ten thousand time steps are calculated for each simulation with a fixed time step of 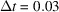 . The temporal evolution of the order parameter for different parameter configurations is depicted in Figs. 1 ▸(*a*) and 1 ▸(*b*). Apart from the initial noise configuration, the simulations differ only in the values of the parameters ε and 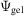 . Both were systematically varied within the intervals 

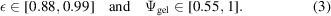

These intervals were chosen with 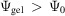 , thus avoiding an arrest of the phase separation before the formation of droplets. We restrict our analysis to large values of ε as for small values of ε the concentration of the dense phase is too low [gray line in Fig 1 ▸(*a*)] to introduce visible changes in the simulations for different values of 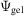 .

**Figure 2:**
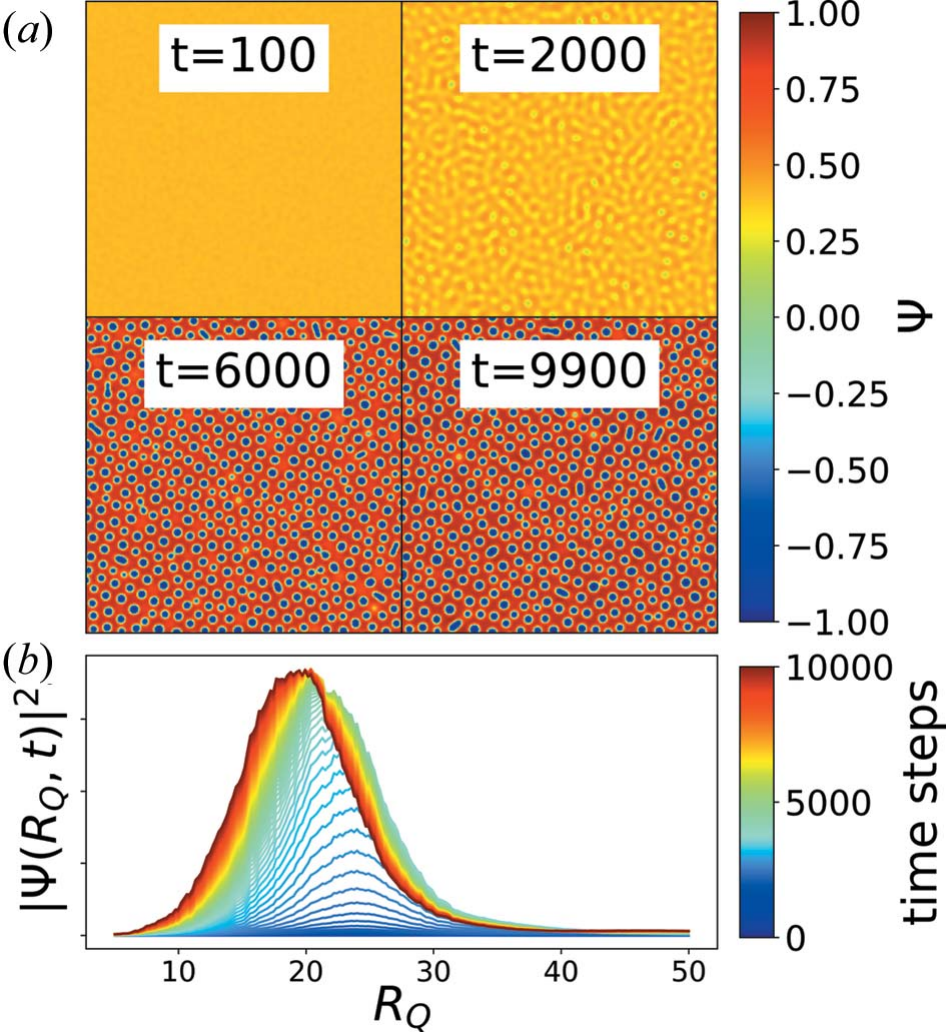
(*a*) Evolution of the real-space configuration of the sample on the 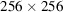 grid with simulation parameters 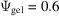 and 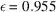 , showing the creation and coarsening of droplets with a low concentration. (*b*) Corresponding temporal evolution of the azimuthally integrated scattering intensity as a function of the absolute value of the distance 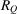 to the origin of the Fourier map. The peak in the intensity profile is shifting towards smaller 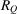 with time, indicating the coarsening of the droplets.

A single simulation run yields a time series of the protein density’s real-space configuration 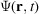 [Fig. 2 ▸(*a*)] which is converted to an X-ray speckle pattern by means of Fourier transform, yielding a 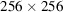 image. The temporal evolution of the square of the azimuthal integration of these Fourier images 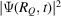 is shown in Fig. 2 ▸(*b*) and is equivalent to the X-ray intensity. We introduce 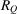 as the distance from the center of the Fourier image in units of pixels. In Fig. 2 ▸(*b*) the occurrence of a peak indicates the phase separation.

The dynamics on a certain length scale in Fourier space can be traced by correlating pixelwise the corresponding intensities with a TTC,

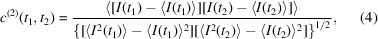

with 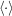 being the average over pixels within a distance 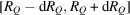 to the center.

The azimuthally integrated intensity profile [Fig. 2 ▸(*b*)] shows the growth dynamics of the droplets by a shift of the peak position towards smaller values of 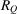 . These dynamics can be observed in the TTC by choosing a value of 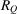 such that it is located at the falling slopes of the peaks in Fig. 2 ▸(*b*), which is 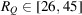 . For our training data, we chose 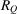 to be an integer in this range, while 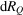 increases linearly from 0.7 at 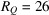 to 3 at 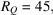 as done in experimental analyses as well to compensate for lower photon counts at higher *Q* values.

### 2.2. Experimental data

**Figure 3:**
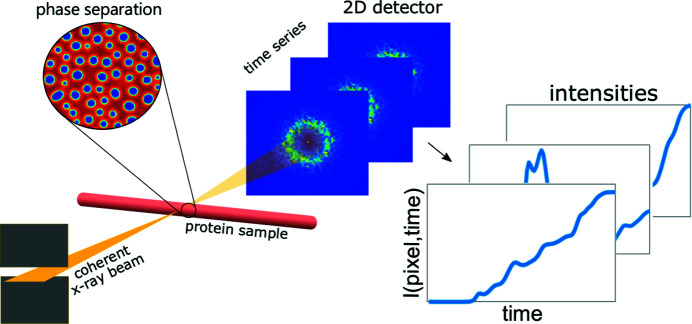
Schematic of the experimental setup. The coherent X-ray beam is scattered from the protein solution during the LLPS yielding a time series of X-ray speckle patterns. A constant *Q* ring is selected in the speckle patterns and the intensities are correlated pixel by pixel for different times.

The XPCS experiments (Fig. 3 ▸) were conducted at the Coherence Applications beamline P10 at PETRA III, DESY, employing an X-ray beam of photon energy 8.54 keV, size 100 × 100 µm and maximum photon density 10^7^ photons s^−1^ µm^−2^ [for further experimental details, see Girelli *et al.* (2021 ▸)]. We measured time series of coherent diffraction patterns that were collected with an EIGER 4 Mpixel detector covering a *Q* range from 3 to 50 µm^−1^. The samples consisted of immunoglobulin G (IgG) with polyethylene glycol (PEG) and NaCl, and were quenched from 37°C to six different quench temperatures below the binodal line. Further details about the sample preparation can be found in the work of Da Vela *et al.* (2017 ▸).

In the experiment, 4000 frames were recorded for every quench temperature. The sample was illuminated for 0.02 s followed by 0.1 s where the shutter was closed such that the measurement covers a time range of 4000 × 0.12 s = 480 s. The experimental TTCs were calculated similarly to the TTCs from the simulation using equation (4). Regarding the temporal shift of the integrated intensity profiles as a function of *Q*, the falling edge can be restricted to 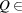 [7.5, 10] µm^−1^. The contrast of the experimental TTCs is lower than that of the simulated ones where we assume completely coherent scattering and detection. This issue is addressed by normalizing the experimental TTCs to the speckle visibility which is determined by the values of the pixels adjacent to the diagonal of the experimental TTC, as the values directly on the diagonal are distorted by shot noise (Inoue *et al.*, 2012 ▸).

### 2.3. Data preparation and auto-encoding

For the training data, 12 000 pairs of the parameters ε and 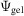 were sampled from a uniform distribution within the intervals. To increase the simulation robustness against the random initial conditions, TTCs from five simulations with different initial noise were averaged. Thus, 5 × 12 000 = 60 000 simulations were performed in total, which took 7 h on two NVIDIA 2080 Ti graphic cards. TTCs were calculated for 20 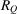 ranges, so that the training data consisted of 20 × 12 000 = 240 000 TTCs.

**Figure 4:**
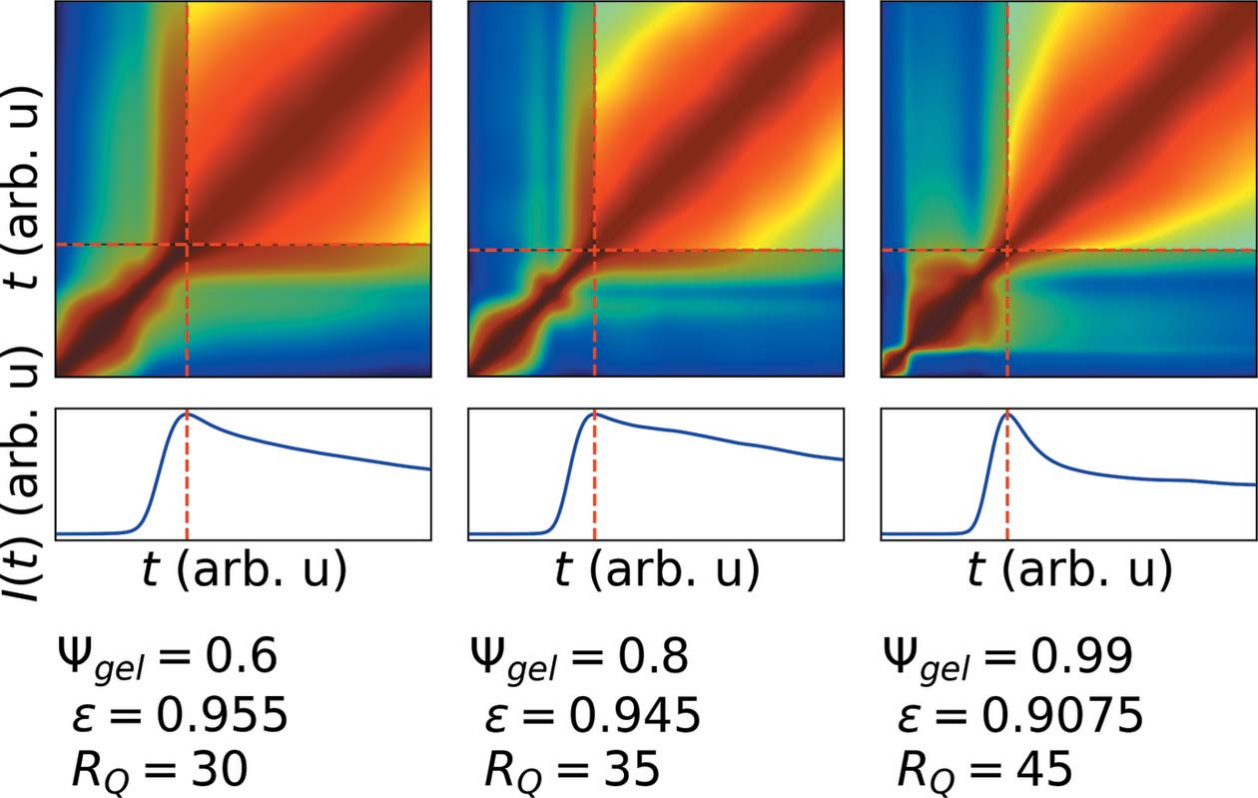
The simulated TTCs show a broad feature at early times (lower-left corner of the TTCs). In the second row, the mean intensity of the respective *Q* range is shown as a function of time. The maximum of this intensity curve defines the point where the TTCs are cropped.

All simulated TTCs display a broad, slow part in the beginning (Fig. 4 ▸) which is absent in the experimental data. This part is associated with the still homogeneous phase at early times before the onset of density differences [Fig. 2 ▸(*a*), *t* = 2000]. Approaching smaller values of 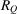 this slow part becomes even more pronounced.

These broad parts are eliminated by cropping the TTCs. The maximum of the intensity in the associated *Q* rings is well defined for both simulated and experimental TTCs and is chosen to be the time where the TTCs are cropped. By doing this, the early part of the phase separation is removed from the TTC, such that only the coarsening stages of simulated and experimental TTCs are compared. The cutting procedure is illustrated in Fig. 4 ▸ for three simulated TTCs. For different models, this preprocessing via cropping needs to be adapted to remove, for example, well known deficiencies from simulated TTCs or make sure that the timescales of simulation and experiment are comparable. We derive the latter from the facts that the intensity curves show similar behavior within the coarsening stage for both experimental and simulated data and the TTCs show the same overall features in the whole measurement time (Girelli *et al.*, 2021 ▸). The cropped TTCs are finally scaled down to a 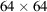 resolution.

Instead of matching raw TTCs between the experiment and the simulations, we compare their encoded representations via an auto-encoder (Rumelhart *et al.*, 1986 ▸). The auto-encoder network (Starostin, 2022 ▸) was trained with the simulated TTCs, encoding them in 32-dimensional vectors 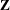 . For the encoder, the first three layers of the pretrained ResNet-18 model (He *et al.*, 2016 ▸) were used as a feature extractor, followed by adaptive average pooling with an output size of 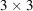 and a fully connected network. The decoder architecture follows the work of Hou *et al.* (2017 ▸).

**Figure 5:**
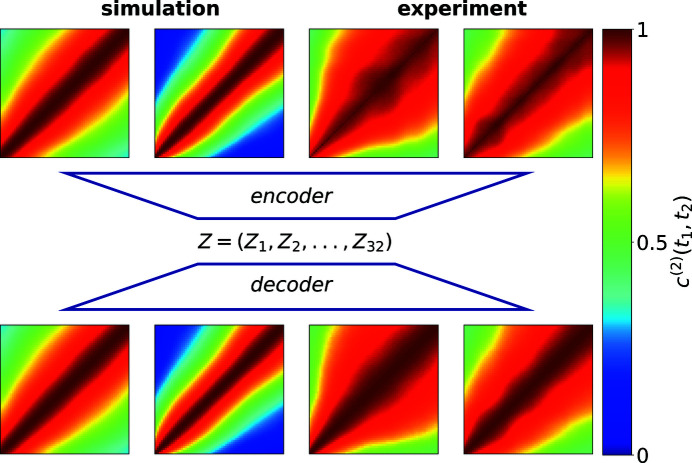
Performance of the auto-encoder on simulated and experimental TTCs. The encoder network encodes the TTCs into a 32-dimensional vector from which the decoder network restores the TTC. On the experimental data, this procedure reduces the experimental TTC to the main dynamic components, enabling a comparison with simulated TTCs.

**Figure 6:**
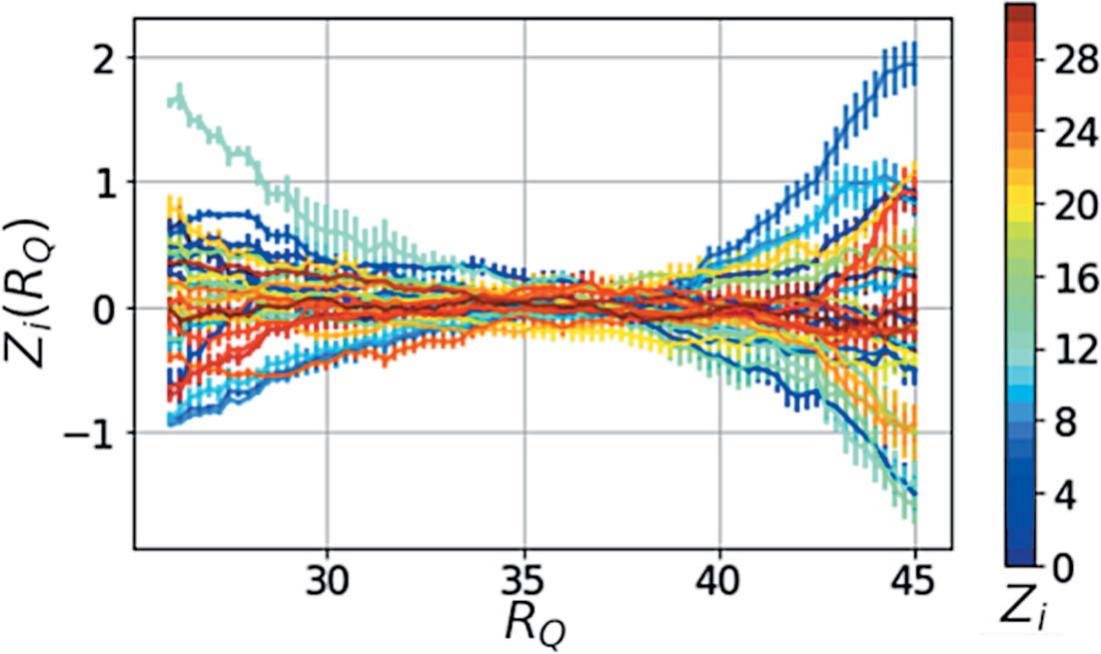
Example of encoded vector components’ 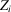 dependence on 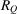 for a simulation with 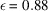 and 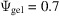 . The error bars result from averaging over ten simulations with different initial noise.

The performance of the auto-encoder network on simulated and experimental data is shown in Fig. 5 ▸, from which one can see that the network is capable of reducing the experimental TTCs to the main dynamic components. Thus, the encoded representations allow comparison of TTCs in a more reliable and faster way. If a series of TTCs from the same simulation but at different values of 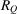 is created, the entries of the 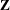 vector change continuously (Fig. 6 ▸) which enabled us to interpolate TTCs for arbitrary values of 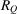 in between the selected values.

### 2.4. Results

Our goal is to develop tools for a fast classification of experimental TTCs in the context of different models. We approach this task via matching encoded TTCs from measurements at different quench temperatures with encoded TTCs from simulations of our simplified model. During this matching process, we find the quench temperature dependence of the two simulation parameters and the values of the three additional calibration parameters 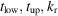 , where 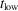 and 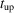 are the times at which the experimental TTCs are cropped such that they match the timescale of the simulation. 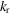 denotes the factor for converting the *Q* range in units of pixels, which were used in the simulation, to units of inverse micrometre as they were used in the experiment via 

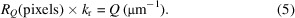

A differential evolution (Price, 1996 ▸) based algorithm was employed for this matching process. For each of the six experimental measurements, TTCs were calculated at five different *Q* values, 

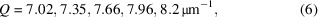

such that we obtain a set of 30 experimental TTCs. The differential evolution algorithm optimizes the mean dot product *D* between the normalized encoded representations of 30 experimental TTCs and 30 simulated TTCs from the training data set with respect to 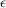 , 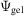 , 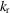 , 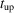 and 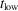 : 

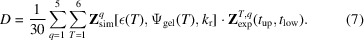




The choice of boundaries for the calibration parameters was based on the assumption that the duration of the experiment is longer than that of the simulation, and that the chosen *Q* range [7.02; 8.2] µm^−1^ lies within the used 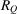 range for the simulations. Knowing that ε is proportional to 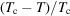 , we apply additional boundaries to consider only those solutions where the average value of ε is decreasing as a function of experimental quench temperature.

During the fitting procedure, the experimental TTCs were cropped and encoded for each generated combination of the parameters. A caching technique was used to accelerate these calculations. The encoded vectors of the simulated TTCs were taken from the training data and interpolated on the basis of the generated calibration coefficient 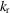 . After 1500 iterations the result of the fit converged to 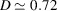 .

**Figure 7:**
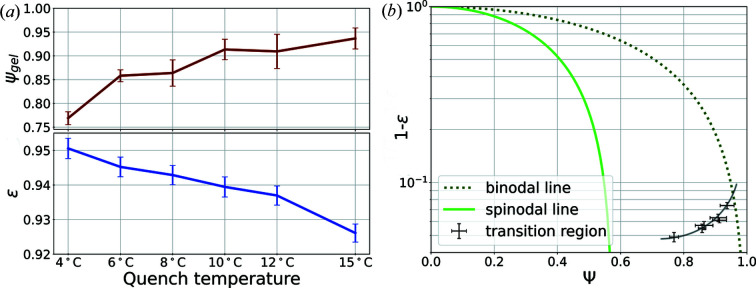
(*a*) Fitted parameters ε and 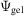 as a function of quench temperature. (*b*) Phase diagram derived from the free energy density used in the simulation displaying the spinodal and binodal line. The points represent the predictions of the neural networks for the experimental TTCs and mark the region of the gelation (guide to the eye) in the framework of the simulation.

Fig. 7 ▸(*a*) shows the averaged outcomes for the simulation parameters 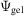 and ε. The rise in 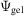 for shallower quenches indicates that the gelation is occurring at higher concentrations at these temperatures. The ( 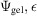 ) pairs were integrated into a phase diagram [Fig. 7 ▸(*b*)] which displays the spinodal and binodal lines as determined from the Landau free energy [equation (1)]. The predictions of the neural network enable us to estimate the corresponding gel line [line in the lower-right part of Fig. 7 ▸(*b*) as a guide to the eye]. This line bends towards the spinodal line, resulting in lower concentrations of the dense phase for deeper quenches. Such a behavior of the gel line in the coexistence region has also been observed for the LLPS of BSA-YCl_3_ (Da Vela *et al.*, 2020 ▸), lysozyme (Cardinaux *et al.*, 2007 ▸) and γ-globulin (Da Vela *et al.*, 2017 ▸).

Averaging over the 30 outcomes for the calibration parameters yields

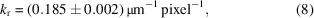




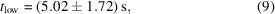




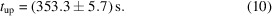

A further validation of these results with respect to ε and 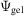 for the experimental TTCs is difficult as neither parameter is directly accessible in the experiment.

## 3. Discussion and conclusion

In conclusion, we successfully trained an auto-encoder neural network with TTC data originating from Cahn–Hilliard simulations. This auto-encoder was used to extract the main dynamic components out of TTCs originating from XPCS experiments of LLPS in a model protein solution of IgG. We used a differential evolution based algorithm for matching encoded TTCs from the experiment and the simulation and determine thereby the parameters necessary for connecting length and timescales of simulation to experiment. The simulation parameters of the classified experimental TTCs were used to construct a phase diagram with the experimental data indicating the position of a gel line.

The training of neural networks based on dynamical simulation data is challenging. The Cahn–Hilliard model employed here, including the gelation, is rather simplistic and uses two labeling parameters only. However, we emphasize the possibility of extending this methodology to more complicated models capable of making strong predictions and eventually also benchmarking of theoretical models based on very large XPCS data sets, possibly by including the kinetic information in the model as well. This is a first step towards handling large amounts of XPCS data on short timescales and will be of general interest not only for protein dynamics but for material science aspects of spinodal decomposition.
